# Neural model of biological motion recognition based on shading cues

**DOI:** 10.1186/1471-2202-16-S1-P81

**Published:** 2015-12-18

**Authors:** Leonid A Fedorov, Martin A Giese

**Affiliations:** 1 Section f. Computational Sensomotorics, Dept. of Cogn. Neurology, CIN/ HIH, University Clinic Tuebingen, Tuebingen, Germany

## 

Point-light or stick-figure biological motion stimuli, due to the absence of depth cues, can induce bistable perception, where the walker is perceived as heading in two alternating directions [1,2]. Psychophysical studies suggested an importance of depth cues for biological motion perception [3]. However, neural models of biological motion perception so far have focused on the processing of features that characterize the 2D structure and motion of the human body [4,5]. We extend such models for the processing of shading cues in order to analyze the three-dimensional structure of walkers from monocular stimuli.

## Model

**Figure 1:**
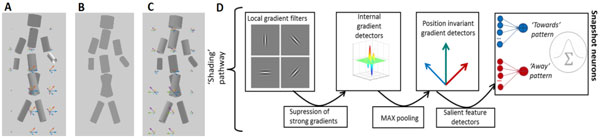
**A. Snapshot from a walker stimulus, rendered from a -45° side view**. Vectors indicate internal luminance gradients, extracted by the internal gradient detectors of the model. **B**. Silhouette stimulus without shading cues is ambiguous and compatible with view angles ±45°. **C**. Snapshot and internal shading gradients for +45° side view. **D**. 'Shading pathway'. After suppression of strong boundary gradients, internal luminance gradients are extracted, using a hierarchy of neural detectors similar to a convolutional network. At the highest level is formed by 'snapshot neurons', RBF units that have been trained with keyframes from 3D walker movies, which are embedded in a dynamic neural field.

As extension of a learning-based neural model [4], we add a 'shading pathway' that computes the internal contrast gradients that vary with the 3D view of the walker, even if the silhouette information remains identical (Figure 1A-C). The model exploits physiologically plausible operations. After suppression of strong external luminance gradients caused by the boundaries of the silhouette, internal luminance gradient features are extracted by a hierarchy of neural detectors. These gradient features, combined with the shape features extracted by the form pathway of the model in [4], are used as input for 'snapshot neurons', RBF units that detect 3D body shapes (Figure 1D). These model neurons are embedded within a two-dimensional recurrent neural field [6] that jointly represents the sequential temporal structure of the stimulus and the view of the walker.

## Results

The neural field dynamics reproduces perceptual multi-stability and spontaneous perceptual switching between stimulus views, observed for silhouette stimuli in psychophysical experiments [1,2]. It also reproduces the disambiguation by addition of shading information and a new perceptual illusion, which illustrates a lighting-from-above prior in the processing of biological motion stimuli.
